# Resultado Evolutivo de Infartos Agudos do Miocárdio em Cinco Regiões Geográficas Brasileiras ao Longo de Duas Décadas

**DOI:** 10.36660/abc.20200563

**Published:** 2020-11-01

**Affiliations:** 1 Universidade de São Paulo Faculdade de Medicina Instituto do Coração São PauloSP Brasil Instituto do Coração - Faculdade de Medicina da Universidade de São Paulo, São Paulo, SP – Brasil

**Keywords:** Doenças Cardiovasculares/complicações, Infarto do Miocárdio, Mortalidade, Morbidade, Epidemiologia, Administração em Saúde Pública

Este é um método de pesquisa atual para avaliar o desfecho de doenças ao longo de décadas, principalmente nas doenças mais prevalentes, como o infarto do miocárdio, um problema de saúde pública com impacto significativo na morbidade e mortalidade da população. Além de evoluir ao longo do tempo, as condições locais também podem contribuir para diferenças de desfecho, principalmente em países com dimensões continentais como o Brasil, bem como com grandes populações (206.081.432 habitantes em 2016; 210.147.125 habitantes em 2019).^1^ Outras variáveis epidemiológicas, como idade, sexo, acesso a tratamento, comorbidades, etc., fazem parte do espectro contínuo da atenção à saúde e dos fatores de risco para doenças cardiovasculares.

No presente estudo^2^ os autores avaliaram dados de mortalidade do Sistema de Informação sobre Mortalidade em cinco regiões geográficas brasileiras entre 1996 e 2016. Os pesquisadores corrigiram dados obtidos de atestados de óbito ajustando-os para: a) causas mal definidas de morte; b) causas básicas inespecíficas ou incompletas (*garbage codes*) sem sentido para um estudo de causalidade; c) correção por sub-registro ou notificação. A análise foi realizada em uma série temporal com regressão linear segmentada.

Inconsistências refletem limitações nas instalações e recursos de tratamento, no diagnóstico e nos detalhes operacionais na coleta, processamento e notificação de dados nos atestados de óbito. Esforços para melhor qualificação dos dados de saúde são um objetivo constante na atenção à saúde e continuamente estimulados nos diferentes níveis de organização do Sistema^3^ coleta e análise de dados.^4^^,^^5^

Os resultados do estudo demonstraram diferenças na qualidade dos dados examinados entre as capitais e algumas cidades do interior. Houve tendência de diminuição da mortalidade por infarto do miocárdio. No entanto, essa queda não foi homogênea em todas as regiões do país no período do estudo. Na região Nordeste não foi este o caso.

Além das diferenças regionais, surgiu uma diferença adicional em relação ao sexo: houve um aumento da mortalidade em mulheres na região Nordeste entre 2002 a 2006. As apresentações fisiopatológicas, clínicas e os desfechos podem ter características específicas nas mulheres em relação aos homens.^6^^,^^7^ Em alguns estudos foi sugerido que o acesso ao tratamento pode ser otimizado.^8^^,^^9^

Algumas outras variáveis relacionadas ao desfecho estão fora do escopo dessas investigações, conforme reconhecidos pelos autores, como hipertensão, obesidade, diabetes, tabagismo, hipercolesterolemia, histórico familiar de doença cardiovascular e infarto do miocárdio. No entanto, elas não chegam a atingir um ponto de aprendizado com a contribuição dos achados deste estudo.

Os autores concluíram que, apesar de uma tendência de queda na mortalidade por infarto do miocárdio em diferentes regiões do Brasil, essa queda foi heterogênea no decorrer desta pesquisa e em relação ao sexo. Significa que há avanços a serem trabalhados neste campo da saúde.
