# Supplementary material for: Resultado Evolutivo de Infartos Agudos do Miocárdio em Cinco Regiões Geográficas Brasileiras ao Longo de Duas Décadas
Source: Arq Bras Cardiol. 2020 Nov 1;115(5):860–1. [Article in Portuguese] doi: 10.36660/abc.20200563 (PMC8452189; doi:10.36660/abc.20200563)
Supplement: Translation

# Translation: Evolving Outcome of Acute Myocardial Infarctions in Five Brazilian Geographic Regions Over Two Decades

**Affiliations:** 2 São PauloSP Brazil Instituto do Coração - Faculdade de Medicina da Universidade de São Paulo, São Paulo, SP – Brazil

**Keywords:** Cardiovascular Diseases/complication, Myocardial Infarction, Mortality, Morbidity, Epidemiology, Public Health Administration

It is a current research method to evaluate the outcome of diseases over decades, mainly in most prevalent diseases such as myocardial infarction, a public health issue with a significant toll in morbidity and mortality of the population. In addition to evolving over time, local conditions may also contribute to differences in outcome, mainly in countries with continental dimensions as Brazil is, as well as with large populations (206.081.432 habitants in 2016; 210.147.125 habitants in 2019).^1^ Other epidemiologic variables, such as age, sex, access to treatment, co-morbidities, etc. – are part of the continuous spectrum of health care and risk factors for cardiovascular diseases.

In the current study^2^ authors evaluated mortality data from a National Database System on Mortality in five geographic Brazilian regions between 1996 and 2016. Researchers corrected obtained from death certificates adjusting for: a) ill defined causes of death; b) codes-garbage without meaning for a causality study; c) correction for under registry or notification. The analysis was made in time series with segmented linear regression.

Inconsistencies would reflect limitations in treatment facilities and resources, in diagnosis and operational details in data collecting, processing and reporting in the deaths certificates. Efforts for better qualifying health data is a constant drive in health care and is continuously stimulated at the different levels of organization of the system,^3^ data collection and analysis.^4^^,^^5^

The results of the study demonstrated differences in the quality of examined data between State capitals and some cities in the countryside. There was a tendency to decrease in mortality due to myocardial infarction. However, such a decrease was not homogeneous in every region of the country in the study period. In the Northeast region that was not the case.

In addition to the regional differences, an additional difference emerged relative to sex: there was an increase in mortality in women in the Northeast region between 2002 to 2006. Pathophysiological, clinical presentations, and outcome may have specific characteristic in women relative to men.^6^^,^^7^ In some studies, it was suggested that access to treatment might be optimizes.^8^^,^^9^

Some other variables related to outcome are out of the scope of this investigations as the authors recognized such as hypertension, obesity, diabetes, smoking, hypercholesterolemia, family history of cardiovascular disease and myocardial infarction. However, they are not to the point of learning with the contribution of the findings of this study.

The authors concluded that in spite of a tendency in decrease in mortality due to myocardial infarction in different regions of Brazil, such a decrease was heterogeneous in the time course of this research and relative to sex. It means that there is progress to be worked out in this field of health care.
